# A randomised controlled trial linking mental health inpatients to community smoking cessation supports: A study protocol

**DOI:** 10.1186/1471-2458-11-570

**Published:** 2011-07-17

**Authors:** Emily AL Stockings, Jennifer A Bowman, John Wiggers, Amanda L Baker, Margarett Terry, Richard Clancy, Paula M Wye, Jenny Knight, Lyndell H Moore

**Affiliations:** 1 University of Newcastle, University Drive, Callaghan, NSW, 2308, Australia; 2 Mental Health and Substance Use Service (MHSUS), Level 4 McAuley Centre, The Mater Hospital, Edith Street Waratah, NSW, 2298, Australia; 3 Centre for Brain and Mental Health Research (CBMHR), Level 5 McAuley Centre, The Mater Hospital, Edith Street Waratah, NSW, 2998, Australia; 4 Hunter New England Population Health (HNEPH), Wallsend Health Services, Longworth Avenue Wallsend, NSW, 2287, Australia

## Abstract

**Background:**

Mental health inpatients smoke at higher rates than the general population and are disproportionately affected by tobacco dependence. Despite the advent of smoke free policies within mental health hospitals, limited systems are in place to support a cessation attempt post hospitalisation, and international evidence suggests that most smokers return to pre-admission smoking levels following discharge. This protocol describes a randomised controlled trial that will test the feasibility, acceptability and efficacy of linking inpatient smoking care with ongoing community cessation support for smokers with a mental illness.

**Methods/Design:**

This study will be conducted as a randomised controlled trial. 200 smokers with an acute mental illness will be recruited from a large inpatient mental health facility. Participants will complete a baseline survey and will be randomised to either a multimodal smoking cessation intervention or provided with hospital smoking care only. Randomisation will be stratified by diagnosis (psychotic, non-psychotic). Intervention participants will be provided with a brief motivational interview in the inpatient setting and options of ongoing smoking cessation support post discharge: nicotine replacement therapy (NRT); referral to Quitline; smoking cessation groups; and fortnightly telephone support. Outcome data, including cigarettes smoked per day, quit attempts, and self-reported 7-day point prevalence abstinence (validated by exhaled carbon monoxide), will be collected via blind interview at one week, two months, four months and six months post discharge. Process information will also be collected, including the use of cessation supports and cost of the intervention.

**Discussion:**

This study will provide comprehensive data on the potential of an integrated, multimodal smoking cessation intervention for persons with an acute mental illness, linking inpatient with community cessation support.

**Trial Registration:**

Australian and New Zealand Clinical Trials Registry ANZTCN: ACTRN12609000465257

## Background

Persons with a mental illness are one of the largest remaining groups of smokers, comprising an estimated 32% of the total smokers in Australia [1]. Consistently high rates of smoking have been found among the mentally ill in Australia and internationally, ranging from 36% in community samples to above 90% among inpatients with psychosis [2-5]. Smokers with a mental illness are also more nicotine dependent [6], more likely to smoke unfiltered cigarettes [7] and less likely to quit than smokers in the general population [8-10]. Consequently, smokers with a mental illness have a significantly reduced life expectancy and are more likely to die from smoking related disease including cancers, cardiovascular disease, respiratory disease and stroke [11,12].

Evidence for the effectiveness of multimodal smoking cessation interventions utilising combined pharmacological and psychosocial support is well established for smokers in the general population [13-15]. Recent evidence suggests that smokers with a mental illness have similar levels of motivation to quit as the general population [16-18] and smoking cessation intervention strategies can be equally effective among this group [19-21]. Multimodal smoking cessation interventions have been found to be effective among US veterans with Post-Traumatic Stress Disorder (PTSD) [22], depressed smokers [23], and in smokers with schizophrenia [24].

General hospitals can provide a base for the initiation of effective smoking cessation interventions [25-28]. Abstaining from tobacco during hospitalisation has been associated with higher abstinence rates at 6 months post discharge [29]. The recent introduction of smoke-free policies in Australian mental health facilities [30] provides the opportunity for smokers to temporarily abstain from cigarettes in a supportive environment, and may facilitate sustained cessation attempts upon discharge [2,31]. Hospitalisation within a smoke free mental health facility has been found to increase patients' desire to quit smoking during admission [4,31], and has been associated with a reduction in daily cigarette consumption from admission to discharge [2].

However, the limited data available indicate that smoke-free policies in mental health facilities appear to have had little effect on long term cessation [25], a finding suggested to be due in part to the lack of coordination between inpatient and community smoking cessation treatment [32,33]. Systematic reviews show that by better integrating inpatient smoking care with post discharge cessation support, long term quit rates are increased among general hospital patients [27,28]. However, in the context of mental health services, low levels of smoking cessation treatment have been found in both inpatient and community-based psychiatric services [34-36], and as a consequence, many smokers return to pre admission smoking levels upon discharge from a mental health hospital [4,31,37]. The limited provision of smoking cessation treatment in community-based psychiatric services, to which many patients are likely to be referred upon discharge, highlights the need for integrated post-discharge smoking cessation treatment for smokers with a mental illness [32,33].

Although an Australian randomised control trial of outpatients with psychosis reported that a multimodal smoking cessation intervention was effective in reducing smoking rates [21], the authors are not aware of any published studies that have examined the effectiveness of integrating inpatient smoking cessation care with community cessation support for individuals with a mental illness. This study is the first of its kind internationally to test, via randomised controlled trial, the feasibility, acceptability and efficacy of integrating inpatient smoking care with post discharge ongoing, multimodal smoking cessation treatment for persons with an acute mental illness. This paper describes the methodology to be employed in the conduct of this trial.

## Methods/Design

### Study aim

The aim of this study is to test a multimodal smoking cessation intervention, linking hospital inpatient care (in a smoke free mental health facility) with post-discharge community cessation support for smokers with a mental illness. This study aims to evaluate the feasibility, acceptability and efficacy of the integrated intervention to reduce smoking behaviour and encourage quitting post-discharge. This study will also provide a detailed evaluation of the uptake and use of the study intervention components, including the cost of the intervention.

### Study Design and Setting

This study will employ a single-site prospective randomised controlled study design, and will be reported in accordance with the requirements of the CONSORT statement [38]. It will be conducted at a large regional inpatient mental health facility located in the Hunter New England region of New South Wales (NSW), Australia. Inpatients will be recruited from three units within the facility (two general adult units and one dual diagnosis unit), with a total of 66 beds, with three other units excluded from this study (two emergency psychiatric care units and one geriatric unit). The majority of the study intervention will be delivered in the community setting, upon participants' discharge from hospital and contact between participants and project officers will occur via telephone and mail.

**Figure 1:**
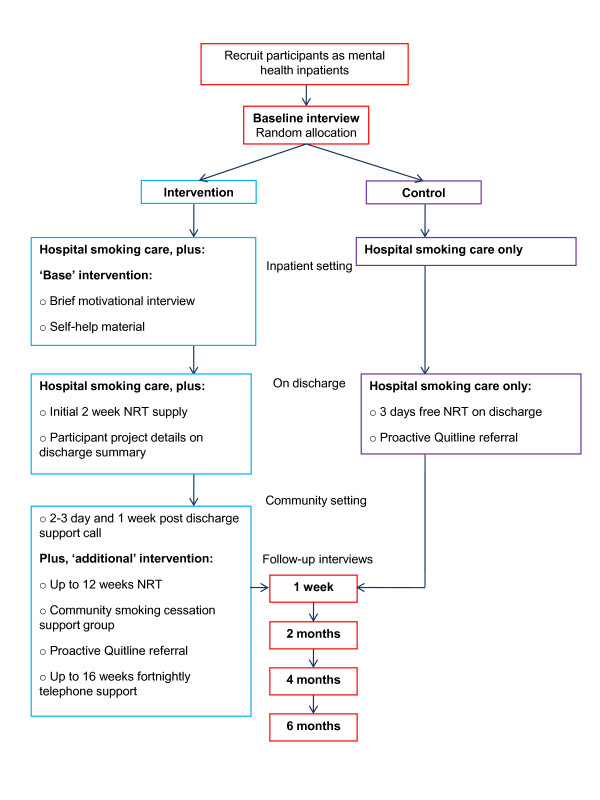
**Study Design**.

Figure 1 shows the study design. Mental health inpatients who report being current smokers will be approached to participate. Participants will be randomly allocated to intervention or control conditions. A permuted block randomisation approach will be used so that the distribution of participants by diagnosis (psychotic; non-psychotic) across treatment conditions will be maintained regardless of the final sample size [39,40].

Inpatients allocated to the intervention condition will receive a brief motivational intervention from the research staff, and will be offered a range of psychosocial and/or pharmacological supports for up to 16 weeks post discharge. An initial two week supply of nicotine replacement therapy (NRT) is provided at discharge, and details of the patient's participation in the study will be added to their electronic discharge summary, for the information of health professionals providing post-discharge treatment.

Patients allocated to the control condition will receive standard hospital smoking care only, which may include provision of up to 3 days free NRT and a referral to Quitline upon discharge. Baseline data will be collected during the initial face to face interview in the inpatient setting by trained project officers.

Follow-up outcome data will be collected by blind interviewers independent of the study, via telephone interview at four time points following discharge: one week, two months, four months and six months.

This project has received ethics approval from the Hunter New England Human Research Ethics Committee, HREC reference no: 08/04/16/5.10, and the University of Newcastle Human Research Ethics Committee reference no: H-2008-0191.

### Participants and research eligibility

Approximately 200 smokers will be recruited to the study (100 in each condition). Written, informed consent will be obtained from each potential participant before commencement of the baseline interview.

#### Inclusion criteria

Participants will be required to be at least 18 years of age and to self report being a current or occasional smoker upon admission to hospital. Participants will also be required to have a contact telephone number and address at the time of recruitment, and be judged by clinical staff to be physically and psychologically capable to complete the face to face baseline interview. Any person who presents severe psychological distress during the baseline interview will be referred to clinical staff and the interview will cease. Eligibility will be reassessed after these patients have stabilised.

#### Exclusion criteria

Patients will be excluded if they are not current smokers, are younger than 18 years of age, do not have a current contact telephone number or address, are non English speaking, or if their current physical or mental wellbeing is judged by clinical staff to be too unstable to participate.

### Recruitment and Allocation

Recruitment of participants will be ongoing over, approximately, a one year period. A systematic daily (weekdays only) review of current patients within each unit will be undertaken in conjunction with clinical staff, with advice provided to project staff regarding those patients deemed to be sufficiently stable to be approached on that day. The study will aim to approach all patients at some time during their stay, establishing the smoking status of those patients who agree to speak with project staff. If patients report being a non or ex-smoker, they will be thanked for their time, and the interview will cease. Eligible patients will be offered participation in the project, provided with the information statement about the research, and written informed consent will be obtained. Participants will complete a baseline interview with the project officer (up to 1 hour duration).

#### Allocation

Prior to commencement of recruitment, a random allocation sequence will be generated using SPSS through consultation with an independent statistician not actively involved in the project. The randomisation will follow a permuted block design, restricted to blocks of 10, and stratified by diagnosis (psychotic; non-psychotic) to ensure an even distribution of participants within and between treatment conditions [39,40]. The random allocation sequence will be stored with a research assistant independent of the recruitment process, and all project officers involved in recruitment and follow-up data collection will be kept blind to the sequence. According to the order of the random allocation sequence, the research assistant will place small cards indicating the treatment condition ("Intervention" or "Control") inside sealed, security envelopes, displaying the sequentially ordered participant identification code on the exterior. Following completion of the baseline interview, the project officer will provide the participant with the subsequent envelope in sequence, and the envelope will be opened by the participant at the conclusion of the baseline interview.

### Intervention condition

In addition to hospital smoking care, the participants randomised to the intervention condition will be provided with a 'base' intervention component, comprising a brief motivational interview and smoking cessation self-help material in the inpatient setting. Participants will also be offered the following 'additional' components of the intervention, to occur post discharge: up to 12 weeks of ongoing NRT; a proactive Quitline referral; and a referral to community smoking cessation support groups. Upon discharge, participants will receive an initial two week supply of NRT, and their participation in the study will be recorded on the hospital discharge summary and sent to relevant treating practitioners in the community. Participants will additionally receive supportive phone contact at three days, and one week post hospitalisation, and the delivery of the elected intervention components will commence. Subsequently, participants electing any of the 'additional' intervention components will concurrently receive up to 16 weeks of further fortnightly telephone support.

#### Intervention content

The smoking cessation intervention focuses on the adoption of multiple, evidence based cessation strategies to assist with smoking reduction and abstinence, at the participant's discretion [14,15]. Quitting smoking will be the focus of the intervention. However, a harm minimisation approach will also be employed, through encouraging a reduction in the number of cigarettes smoked per day [41-44]. At the completion of the baseline interview, and following allocation to the intervention condition, participants will receive:

#### 'Base' Intervention component

##### a) Smoking cessation self-help material

Participants will be provided with a clear, plastic folder containing a pamphlet and workbook targeted at smokers with a mental illness developed by a state government provided, proactive telephone smoking cessation support service 'Quitline' [45,46], a smoking and mental health factsheet developed by a national mental health charity organisation 'SANE' [47], and a one page document (developed by the research team) with instructions on how to effectively use NRT, and how to manage nicotine withdrawals and NRT side effects [48].

##### b) Brief motivational interview

The project officer will conduct a brief (5-10 minutes) motivational interview by guiding the participant through a series of topics designed to motivate the participant towards positive health behaviour change, including: positives and negatives of smoking and quitting, importance and confidence in quitting, and health and financial costs of smoking [49].

Immediately upon discharge from hospital, intervention participants will receive:

##### c) Initial NRT supply

To allow for continuation of inpatient and community smoking cessation treatment, an initial two week supply of NRT will be provided to the patient at discharge. Subsequent NRT supplies are delivered in the community setting (below).

##### d) Study participation recorded on discharge summary

The project officer will provide brief information regarding the participants' engagement in the trial into the area health service's online discharge summary system. This information will be communicated to GPs and other relevant health professionals upon the participant's discharge from hospital, in order for these services to support the participants' ongoing care, and for this study, their quit attempt in the community setting [27,28].

Additionally, participants allocated to the intervention condition will be offered the following supports to commence upon discharge:

#### 'Additional' intervention component

##### e) NRT

Participants will be offered up to 12 weeks free NRT [50-52]. An NRT protocol for this trial will be developed based on a combination NRT algorithm [53], the area health services' NRT protocol [30] and the product disclosure information for the NRT provided in this trial. Patch (21, 14 and 7 mg), lozenge, gum (2 and 4 mg), and inhaler (10 mg) will be prescribed according to the study NRT protocol, and based on a nicotine dependence assessment [54] and patient preferences. Combination therapy (patch plus adjunctive) will be recommended for all smokers. For those who smoke > 20 cigarettes per day, 2 mg gum or lozenge, or 10 mg inhaler will be offered to manage acute craving. Tapering of NRT dose will not be explicitly advised, but dosage and frequency of use will be closely reviewed during fortnightly telephone support.

On the day of recruitment, a project officer will send an email notification of the NRT prescription to the participant's Chief and Junior Medical Officer should there be any unknown medical concern. An initial 2 week supply of NRT will be provided to the patient at discharge. Subsequent supplies of NRT will be mailed fortnightly at the completion of the fortnightly telephone support call (below), in which daily cigarette consumption, nicotine withdrawal symptoms and patient preferences will be reviewed.

##### f) Community smoking cessation support groups

Referral will be offered to smoking cessation support groups developed in conjunction with allied health staff in local community mental health services, and delivered by staff in those services [55]. A project officer will complete a referral form for the participant and email it to the appropriate community mental health service (based on local government area) upon the participant's discharge from hospital. Group facilitators will contact participants to complete an initial screening interview and provide details of the group program. Groups will run on a rotating basis of one, one hour group for four weeks, and will follow an informational, group-oriented support and skills training format, with no specific psychological or behavioural intervention [56,57]. Content will be tailored to smoking and mental illness, with topics covered including: understanding nicotine addiction and withdrawal, smoking habits and triggers, benefits of quitting, effective use of NRT, illness management and interaction with medications, and quit strategies.

##### g) Proactive Quitline referral

The NSW Quitline (funded by the Cancer Institute, NSW) [45] is a confidential telephone based service designed to help smokers to reduce or quit tobacco smoking. With the consent of participants, the project officer will complete a proactive NSW Quitline referral form at the completion of the baseline interview, and fax to Quit NSW upon the participant's discharge from hospital. The Quitline will call the participant according to the time and day suggested by the participant on the form, within one week of referral. The telephone service typically involves 6 calls, and a follow-up call conducted three months following the participants' elected quit date, with content including: nicotine dependence; quit strategies; relapse prevention; and information on cessation products and services [45].

##### h) Fortnightly telephone support

For all intervention participants, support calls will be conducted by a telephone counsellor at three days, and one week post discharge [58]. If participants elect any of the 'additional' intervention supports, these calls will continue fortnightly for as long as the participant is engaged with the intervention (up to 16 weeks total). Each call will follow a predefined script as developed by the research team. Topics covered include: uptake, usage, problems and effectiveness of intervention supports (NRT, Quitline, community smoking cessation support groups), fortnightly review of NRT dosage, assistance with NRT use, monitoring and managing nicotine withdrawal symptoms, daily cigarette consumption, techniques to improve smoking outcomes, and general psychological support and encouragement. If participants are receiving study NRT, these fortnightly telephone support calls will act as a means for project officers to monitor NRT use, dosage and side effects, and to subsequently mail an appropriate fortnightly supply of NRT.

#### Intervention personnel, recruitment and training

Recruitment will be conducted by a registered Health Psychologist and several project officers with four year undergraduate Psychology degrees. Prior to commencement of recruitment, all recruitment staff will complete the area health services' mandatory 2-day mental health training, concerned with ensuring occupational health and safety when working in the hospital environment. All recruitment staff will be trained by a senior member of the research team and psychologist undertaking recruitment in conducting the baseline interviews and brief motivational interviewing, including the conduct of mock interviews, and sitting in on patient interviews.

The fortnightly telephone support service will be provided by a registered nurse (and undergraduate psychology student), experienced with patient contact and managing patient issues. The interviewer will undergo project specific training and will assist in the development of the support protocols and content.

Outcome data will be collected by blind telephone interviewers, independent of the study, with several years experience in conducting health related telephone based surveys, but no formal qualifications in psychology or social sciences. Interviewers will undergo project specific training where they will be briefed on the aims and methodology of the study. Details regarding project specific issues (including NRT and psychiatric medication) will be provided to ensure interviewers are able to prompt participants if they have any difficulty or confusion regarding outcome questions.

#### Treatment monitoring and fidelity

To ensure integrity of the intervention, members of the research team will have weekly contact with project officers, support call staff and follow-up telephone interviewers to ensure common issues or concerns are dealt with in a consistent and timely manner. The research team will also meet fortnightly with a working group comprising experienced mental health and drug and alcohol clinicians employed at the hospital site to raise and address any issues arising from working within the hospital and to discuss and gain insight into the best management of participants throughout the trial period. The research team will also meet quarterly with a larger advisory group to discuss the aims of the trial, ensure recruitment, follow-up and intervention delivery are occurring to the best standard possible, and to keep abreast of approach, consent and follow-up rates.

### Control condition

Participants allocated to the control condition will receive standard hospital nicotine dependence treatment only. This may include provision of NRT during hospitalisation and, upon discharge, up to 3 days provision of NRT and referral to 'Quitline'. Nicotine dependence treatment is known to be limited and to vary in this setting [36].

### Data collection and measures

#### Baseline

The baseline survey will be administered as a face to face interview by project officers, within the inpatient setting prior to the allocation of treatment condition, and will be of up to 1 hour duration for both groups.

#### Contact information

During the baseline interview, contact details will be obtained directly from the participant, including: home address, contact phone number, living situation, and smoking status of housemates. In order to reduce attrition, participants will also be asked to elect and provide contact details of two 'contact persons' (friends or family members) and for their regular GP or health practitioner (name, phone number and service address) for use by the research team in the event that participants can no longer be contacted and new details need to be obtained [59,60]. Consent will also be gained from the participant for their contact details to be obtained from another Hunter New England Area Health Service providing care, in the event that their contact details change and the research team cannot contact them through other means.

#### Follow-up data collection

Follow-up data will be collected for both groups via blind telephone interview at one week, two months, four months and six months post discharge. To enable subsequent assessment of the effectiveness of the blinding, interviewers will be asked at the completion of the interview to indicate the treatment condition to which they believe the participant was allocated. If any participant experiences an acute phase of their psychiatric disorder during any of the follow-up phone interviews, they will be encouraged to contact their GP, psychologist or community mental health team. Participation will continue as normal unless the participant expresses severe psychological distress and/or they no longer wish to participate in the trial. To minimise attrition, notices will be mailed, one week prior the due date, to remind participants of each upcoming follow-up call [61]. The protocol for follow-up calls will comprise up to four weeks of call attempts, with at least 10 attempts made within the first seven days of the due date, and regular attempts thereafter. At the completion of the six month follow-up interview, a letter will be mailed to participants, notifying them that their participation is complete and thanking them for their contribution to the study.

#### Demographic information

Demographic details including level of education, employment status and whether the participant receives a government support pension will be collected during the baseline interview. At the completion of the baseline interview, additional demographic information will be obtained from the participant's medical record, by a Health Psychologist employed by the service, including participant's full name, medical record number, age, address at time of admission, gender, pregnancy status (if NRT is to be prescribed), marital status, Aboriginal or Torres Strait Islander cultural identification, date and length of current admission, smoking status on admission, nicotine dependence treatment provided during admission, primary and secondary mental health diagnoses, medications for mental and general health conditions, and date of most recent previous admission to the facility (if applicable).

#### Primary outcome measures

The primary outcomes of this trial relate to changes in participants' smoking behaviour. Smoking behaviour will be assessed at each of the four follow-up surveys (one week, two, four and six months) by: daily cigarette consumption; number and duration of quit attempts; and self-reported abstinence. Daily cigarette consumption and quit attempts are recognised and recommended outcome measures which are frequently used in this population [62,63]. When abstinence is reported, it will be verified by exhaled carbon monoxide (CO) levels using a MICRO+ Smokerlyzer, with a cut-off of < 10 ppm [42,63]. CO validation tests will be arranged during the follow-up surveys and will be conducted by a project officer within 3 days of the survey (with smoking status attained again at that time), in a public location convenient to the participant (eg. library) or, if not viable, conducted as a home visit following the institutional safety guidelines.

#### Secondary outcome measures

Secondary outcome measures will include: nicotine dependence, as measured by the Fagerstrom Test of Nicotine Dependence (FTND) [54]; changes in motivation to quit smoking, as measured by the Readiness and Motivation to Quit Smoking Questionnaire [64]; alcohol and other substance use, as measured by The Alcohol Use Disorders Identification Test (AUDIT) [65]; and mental well being, as measured by the Kessler Psychological Distress Scale (K10) [66].

#### Process measures

##### a) All participants

Details regarding participant uptake, use and perceived effectiveness of smoking cessation supports (provided by this trial or elsewhere) will be collected at each of the four follow-up time points (one week, two, four and six months post discharge) for participants in both conditions. In addition, the follow-up telephone interviewers will record data arising from the outcome of each follow-up telephone survey, including the time and date of all call attempts, call outcome (eg. engaged, answering machine, call partially complete, call complete or refusal), length of call, and the interviewer who conducted the call.

##### b) Intervention participants

For participants allocated to the intervention condition, more detailed information regarding uptake, use and effectiveness of the interventions provided by this trial will be collected systematically at each fortnightly telephone support call, including: intervention options elected at baseline, three day call and initial (one week) support call; whether the participant received their previous NRT allocation and details of its contents; usage of NRT, including type, dosage, and amount used per day; problems with and perceived effectiveness of NRT; smoking cessation support group attendance and effectiveness; number, date and effectiveness of calls received from Quitline; and any problems experienced with the intervention options or participation in the project. Additionally, information regarding the process and outcome of these calls will be recorded, including date and time of call attempts, call outcome (eg. engaged, answering machine, call partially complete, call complete or refusal) call duration, interviewer who conducted the call, and the total number of calls received by each participant in the intervention support phase.

##### c) Cost

This trial will provide a detailed evaluation of the costs to deliver the intervention. Cost will be determined by assessing: staff time associated with initial recruitment and follow-up support calls; NRT and other intervention materials (including self-help brochures); and phone and mail costs. Data for the use in calculation of costs will be collected through detailed records of baseline interview length, support call attempts and length, total NRT usage and associated mail costs.

### Sample size and detectable difference

A total of 200 participants (approximately 100 per group) will be recruited to the study (an estimated 70 per group at 6 month follow-up, assuming a 70% follow-up rate). This number is sufficient to conduct intention to treat analyses to examine intervention effectiveness (with 80% power and .01 level significance tests) in terms of differences on the continuous outcome measures (e.g., quit attempts, daily cigarette consumption, nicotine dependence) of the order of 0.51 standardised (effect-size) units, and 4.8 fold increases in point prevalence abstinence (e.g., treatment group, 24% abstinent, vs. comparison group, 5% abstinent). A previous, similar sized multimodal intervention study conducted among a mental health patient population demonstrated statistically significant differences (20% effect size or larger) between groups on continuous outcomes including reduction in daily cigarette consumption and nicotine dependence levels, but not for abstinence [21]. Due to the unique methodology and intensity of intervention employed in the current trial, it is uncertain whether this trial will have sufficient power to detect statistically significant differences in abstinence between the two conditions, however significant differences in other outcomes are achievable. It could be argued, based on the novel design of this trial, larger effect sizes may be achieved, but this remains speculative.

#### Analysis

Data will be analysed using IBM SPSS Statistics for Windows (version 19.0). For the key smoking-related outcome variables, intention-to treat analyses will be conducted, together with subgroup analyses based on patterns of intervention uptake. For these analyses, missing data will be classified nonabstinent or as failing to achieve reduction. Odds ratios and associated confidence intervals (CI) will be reported, with the control group as the reference point (odds ratio = 1.00). For the continuous outcome variables (e.g., daily cigarette consumption), planned comparisons between follow-up points, from repeated-measures analyses of variance (ANOVAs), will be used to examine group differences in patterns of change. As a partial control for the number of statistical tests, the threshold for statistical significance will be set at p < 0.01. Among this population, evidence of satisfactory engagement with post-discharge support services and progressive changes in smoking behaviour are also considered highly desirable outcomes. The projected sample sizes should also be sufficient to allow an examination of correlations between selected participant characteristics and aggregate indices of engagement based on the various process measures.

## Discussion

The research literature indicates no previously published randomised control trials internationally to evaluate the effectiveness of an integrated smoking cessation intervention for mental health inpatients, linking inpatient smoking care with community cessation supports. This multimodal, integrated intervention design has been developed to maximise the likelihood of positive smoking outcomes for mental health patients, and aims to demonstrate the feasibility, acceptability and potential efficacy of linking inpatient smoking care to community cessation support. The study demonstrates many strengths. Firstly, most smoking cessation interventions for persons with a mental illness to date have focused on specific diagnostic groups, particularly, samples with schizophrenia or schizoaffective disorders [33]; and have further required patients to express a willingness or desire to quit for participation [19]. The methodology employed in this trial is particularly unique. No previous studies have directly examined the effect of linking inpatient smoking care to community cessation support for mental health patients, and only very few have been conducted in the general hospital setting [67,68]. In the current trial, we aim to employ a 'real life' approach, working within existing mental health services, systematically approaching patients and determining smoking status, and offering the project regardless of diagnosis or motivation to quit. By offering participation to a heterogeneous sample of diagnostic groups and motivation levels, this trial may demonstrate the effectiveness of providing integrated smoking cessation treatment to mental health inpatients in a systematic manner that may be incorporated into existing mental health settings.

## Conclusions

This manuscript provides a comprehensive description of the methodology to be employed as part of a randomised control trial to examine the feasibility, acceptability and potential efficacy of an integrated smoking cessation intervention for mental health inpatients, linking inpatient smoking care with community cessation support. The successful implementation of this trial will provide strong evidence on which to base judgments regarding the efficacy of this intervention approach.

## Competing interests

The authors declare that they have no competing interests.

## Authors' contributions

ES drafted the manuscript and contributed to study management and recruitment. JB, JW, AB, PW and JK conceived of the study, participated in its design and assisting in drafting the manuscript. MT, RC and LM participated in the study design and clinical coordination of the trial at the hospital site. All authors read and approved the final manuscript.

## Pre-publication history

The pre-publication history for this paper can be accessed here:

http://www.biomedcentral.com/1471-2458/11/570/prepub
